# Implications of Biological Rates of Aging on Healthcare Expenditures and Morbidity

**DOI:** 10.1093/geroni/igab046.2526

**Published:** 2021-12-17

**Authors:** Alix Jean Santos, Xavier Eugenio Asuncion, Camille Rivero-Co, Maria Eloisa Ventura, Reynaldo Geronia, Lauren Bangerter, Natalie Sheils

**Affiliations:** 1 OptumLabs, Minnetonka, Minnesota, United States; 2 OptumLabs, Eden Prairie, Minnesota, United States

## Abstract

Understanding biological aging, which entails impeding the progressive decline of biological systems, is important in enabling older adults to live independently. However, the differences in how individuals evolve as they age suggest that aging is a process that does not progress on a single-dimensional trajectory. Moreover, longitudinal studies of aging that follow a cohort of individuals over the course of several years are commonly limited by cost, attrition, and subsequently small sample size. In this study, we used a variational autoencoder to estimate multidimensional rates of aging from cross-sectional routine laboratory data of 1.4 million Americans of at least 40 years of age, collected from 2016 to 2019. We uncovered four aging dimensions that represent the following bodily functions: 1) kidney, 2) thyroid, 3) white blood cells, and 4) liver and heart. We found that fast agers along these dimensions are more likely to develop chronic diseases that are related to these bodily functions. They also had higher health care expenditures compared to the slow agers. K-means clustering of individuals based on the different aging rates revealed that clusters with higher odds of developing morbidity had the highest cost across all types of health care services. Results suggest that cross-sectional laboratory data can be leveraged as an alternative methodology to understand rates of aging along different dimensions, and analysis of their relationships with future costs can aid in the development of interventions to delay disease progression.

